# Structure, Function, Diversity, and Composition of Fungal Communities in Rhizospheric Soil of *Coptis chinensis* Franch under a Successive Cropping System

**DOI:** 10.3390/plants9020244

**Published:** 2020-02-13

**Authors:** Mohammad Murtaza Alami, Jinqi Xue, Yutao Ma, Dengyan Zhu, Aqleem Abbas, Zedan Gong, Xuekui Wang

**Affiliations:** 1 Department of Crop Cultivation and Farming System, College of Plant Science and Technology, Huazhong Agricultural University, Wuhan 430070, Hubei, China; murtazaalami@webmail.hzau.edu.cn; 2 Department of Crop Science, College of Plant Science and Technology, Huazhong Agricultural University, Wuhan 430070, Hubei, China; jinqi_xue@webmail.hzau.edu.cn; 3 Department of Pharmaceutical Botany, College of Plant Science and Technology, Huazhong Agricultural University, Wuhan 430070, Hubei, China; mayutao0301@163.com; 4 Department of Agronomy and Seed Science and Technology, College of Plant Science and Technology, Huazhong Agricultural University, Wuhan 430070, Hubei, China; zhudengyan@webmail.hzau.edu.cn; 5 The Provincial Key Lab of Plant Pathology of Hubei Province, College of Plant Science and Technology, Huazhong Agricultural University, Wuhan 430070, Hubei, China; aqlpath@gmail.com; 6 College of Plant Science and Technology, Huazhong Agricultural University, Wuhan 430070, Hubei, China; zedangong@webmail.hzau.edu.cn

**Keywords:** *C. chinensis*, continuous cropping, rhizosphere, fungi diversity, composition, structure

## Abstract

Soil types and cropping systems influence the diversity and composition of the rhizospheric microbial communities. *Coptis chinensis* Franch is one of the most important medicinal plants in China. In the current study, we provide detailed information regarding the diversity and composition of rhizospheric fungal communities of the *C. chinensis* plants in continuous cropping fields and fallow fields in two seasons (winter and summer), using next-generation sequencing. Alpha diversity was higher in the five-year *C. chinensis* field and lower in fallow fields. Significant differences analysis confirmed more fungi in the cultivated field soil than in fallow fields. Additionally, PCoA of beta diversity indices revealed that samples associated with the cultivated fields and fallow fields in different seasons were separated. Five fungal phyla (*Ascomycota*, *Basidiomycota*, *Chytridiomycota*, *Glomeromycota* and *Mucoromycota*) were identified from the soil samples in addition to the unclassified fungal taxa and *Cryptomycota*, and among these phyla, *Ascomycota* was predominantly found. FUNGuild fungal functional prediction revealed that saprotroph was the dominant trophic type in all two time-series soil samples. Redundancy analysis (RDA) of the dominant phyla data and soil physiochemical properties revealed the variations in fungal community structure in the soil samples. Knowledge from the present study could provide a valuable reference for solving the continuous cropping problems and promote the sustainable development of the *C. chinensis* industry.

## 1. Introduction

*Coptis chinensis* Franch is a perennial plant, and is also one of the therapeutically most important medicinal plants, and is commonly used in Chinese traditional medicine. The rhizome has a high therapeutic value. Although the plant has a bitter flavor, the rhizome of the plant is being used as a food ingredient, as well as being added to products such as honey and other drinks [1]. The roots alone or in the combination of other medicinal plants are being used to treat human diseases such as diabetes, dysentery, jaundice, acute febrile and supportive infections, seasonal febrile disorders, sore throat, fever and diarrhea [2].

The *C. chinensis* plant is mainly found in eastern Chongqing, western Hubei, northern Hunan, southern Shanxi, Guizhou and other places of China. However, the main products of the plants are being processed in the Shizhu County of Chongqing City and the Lichuan City of Hubei Province. About thirty alkaloids have been extracted from the plants. Among the alkaloids, the berberine, epi-berberine, palmatine, coptisine and jatrorrhizine are the main constituents [3]. The *C. chinensis* plant grows in the shady, damp and cold climate and valleys of 500–2000 m above sea level.

Soil microorganisms play an important role in the ecosystem, and are the key factors associated with soil quality, soil fertility and productivity. Changes in rhizospherice soil microorganisms affect the absorption and transformation of soil nutrients [4,5,6,7]. Consistently, the quantity and species of rhizospheric soil microorganisms are important factors that affect the growth, development and health status of plants. Some studies have reported that continuous cropping affects the rhizospheric soil microbial structure. In response, such alterations further contribute to the aggravation of continuous cropping obstacles. Thus, the relationship between microbial community structure in the rhizospheric soil and continuous cropping obstacles has attracted increasing attention [8,9,10]. Continuous cropping is the cultivating of the same crop in the same soil for many years, and is a vital agriculture issue due to depletion of the soil qualities and additions of harmful pests and soilborne microbial communities resulting in yield decline [11]. Recently, an increasing number of studies have speculated that long-time continuous cropping changed the diversity of microbial communities in soil [12,13].

Soil microbial communities are essential, and play critical roles in soil health and quality, soil organic matter decomposition, nutrient availability and cycling. Both biotic and abiotic factors like cropping system, plant species and soil types change the composition, diversity, structures and function succession of these soil microbial communities found in the rhizosphere of the *C. chinensis* plants [14,15]. Among these soil microbial communities, the fungal communities in terms of abundance, composition, activity and diversity play an important role in improving the ecosystem to guarantee soil quality and the health of crops [16,17,18].

Previous studies on medicinal plant *Rehmannia glutinosa* suggest that monoculture can modify the fungal community in the rhizosphere [18]. In another study, the amount of *F. oxysporum* increased significantly in the rhizosphere of *P. heterophylla* under continuous cropping [19]. Moreover, continuous cropping of *C. chinensis* resulted in a significant and constant decline in the richness and diversity of the soil fungal population [20]. However, to the best of our belief, no studies are comparing the diversity, composition, structure and functions of the rhizospheric fungal communities of *C. chinensis* plants in different seasons and successive years.

In the present study, the composition, diversity, structure and functions of the rhizospheric fungal communities of *C. chinensis* medicinal plants were observed for five years under continuous cropping in two seasons, winter and summer. The study was conducted based on a large amount of data from the next generation sequencing (NGS) platforms, such as Illumina MiSeq, using 18S fungal primers. Further, the physicochemical properties of soil were also determined and correlated with the fungal communities of the rhizosphere of the *C. chinensis* plant.

## 2. Method and Materials

### 2.1. Study Site and Soil Sampling

The soil samples were collected from crop fields of *C. chinensis* plants in Jiannan town, Lichuan City, Hubei province, China (Coordinates: 108°23′–108°35′ E, 30°18′–30°35′ N), at an altitude of 1500 m, where soil types are mostly sandy and clay, the average annual rainfall between 1198–1650 mm and the average yearly temperature is 12.7 °C. The rhizospheric soil sampling (2 mm) was conducted from different fields continuously cropped with *C. chinensis* for one-year (Y1), three-year (Y3), five-year (Y5) and fallow fields (FF), which were not cropped for more than three years over two seasons, winter (15 August 2018) and summer (10 February 2019). Four *C. chinensis* fields were randomly selected for the experiment. These Four fields were continuously cultivated areas, and another four sites selected were fallow fields. One composite rhizosphere sample was taken per field from the roots of 10 randomly selected *C. chinensis* plants. The plant roots were shaken vigorously to separate soil that was not tightly adhering to the roots. In the fallow fields, there were no plants; the soil samples were collected from the top 15 cm of soil. The soil was then transported to the laboratory in iceboxes. In the laboratory, each soil sample was sieved to remove debris and stony materials using a 2 mm sieve. Each sample was appropriately homogenized, and 10 g subsamples of soils were put into the sterilized tubes and stored at −80 °C for DNA extraction, and other soils were saved for an analysis of the physicochemical properties of this soil.

### 2.2. Analysis of Physicochemical Properties

A Mettler-Toledo TE 20 was used to test the soil pH by making a soil suspension with deionized distilled water (1:20 *w*/*v*). The potassium dichromate internal heating method was used to measure the soil organic matter (TOM). The total content of nitrogen (TCN) and phosphorus (TCP) was measured by using a SMARTCHEM 200 Discrete analyzer, and the total content of potassium (TCK) was measured by using the FP series multi-element flame photometer.

### 2.3. DNA Extraction and PCR Amplification

Microbial DNA was extracted from 0.5 g soil samples (dry weight) using the PowerSoil kit (MO BIO Laboratories, Carlsbad, CA, USA) according to the manufacturer’s protocols. The DNA concentration and purification were indicated by a Nano-Drop 2000 UV-vis spectrophotometer (Thermo Scientific, Wilmington, DE, USA). Furthermore, DNA quality was screened by 1% agarose gel electrophoresis. The DNA extract was pooled and kept at −80 °C until being used. The V5–V7 regions 18S rRNA gene of fungi were amplified with primers SSU0817F (5′-TTAGCATGGAATAATRRAATAGGA-3′) and 1196R (5′-TCTGGACCTGGTGAGTTTCC-3′) [21,22] by the thermocycler PCR system (GeneAmp 9700, CA, USA). PCR reactions were conducted using the program: 3 min of denaturation at 95 °C, 27 cycles of 30 s at 95 °C, then 30 s for annealing temperature at 55 °C, 45 s for elongation at 72 °C and a final extension at 72 °C for 10 min. PCR reactions were carried out in triplicate 20 μL mixtures containing 4 μL of 5× Fast-Pfu Buffer, 2 μL of 2.5 mM dNTPs, 0.8 μL of each primer (5 μM), 0.4 μL of FastPfu Polymerase, 0.2 μL BSA and 10 ng of template DNA. The PCR products results were derived from a 2% agarose gel. In addition, the purification was carried out by using the AxyPrep DNA Gel Extraction Kit (Axygen Biosciences, Union City, CA, USA). The products were quantified using QuantiFluor™-ST (Promega, Madison, WI, USA) according to the manufacturer’s protocol.

### 2.4. Illumina MiSeq Sequencing

Purified amplicons were pooled into equimolar and paired-end sequenced (2 × 300) on an Illumina MiSeq-platform (Illumina, San Diego, CA, USA) according to the manufacturer’s protocol.

### 2.5. Processing of Sequencing Data

Raw FASTQ files were quality-filtered by Trimmomatic and merged by FLASH with the following criteria. (i) The 300 bp reads were shortened at any site receiving an average quality score < 20 over a 50 bp sliding window, and discarding the truncated reads that were shorter the 50 bp. Reads containing N-bases are also discarded. (ii) Sequences whose overlap was longer than 10 bp were merged according to their overlap, with any mismatch being no more than two bp. (iii) Sequences in each sample were separated according to barcodes (exactly matching) and primers (allowing two nucleotide mismatching), and reads containing ambiguous bases were removed [23].

Operational taxonomic units (OTUs) cultivation by similarity using Mothur version 1.31.1 with 97% cutoff points and chimeric sequences, were removed by quantitative insights into microbial ecology (QIIME). The taxonomy of each gene sequence was examined against the Silva (SSU123) database with a confidence threshold of 70% [24].

### 2.6. Statistic Analysis

Statistical analyses were conducted using Statistical Product and Service Solutions (SPSS) 18.0 software and the R vegan package. The remaining sequences of all of the samples were rarefied to the same sequencing depth. Principal coordinates analysis (PCoA) of “the Bray–Curtis distances” was performed using the R package “PCOA.” Venn diagrams were generated with the “venerable” package in R. Redundancy analysis (RDA) of multiple correlation variations among environmental variables (TOC, TCN, TCP, TCK, pH and community composition at the phylum level) was carried out by using the “RDA” function, and the environmental factors were fitted with the ordination plots using the vegan package in R with 999 permutations. The differential OTU abundances were calculated by using the R package “DESeq2.” Differential abundance analysis was performed by fitting the generalized linear model with a contrary binomial distribution to the normalized value of each out, and using a Wald test to test the differential abundance. Enriched and depleted OTUs were defined as OTUs with absolute differential abundance > 1.0 and *p* < 0.05.

One-way analyses of variance (ANOVA) with Tukey’s HSD multiple range tests were done for multiple comparisons and the comparison of Pearson correlation coefficients between the soil properties, and the abundances of fungal phyla were all calculated using SPSS v20.0 (SPSS Inc., Chicago, IL, USA). For alpha diversity, all analyses were based on the OTU clusters with a cutoff of 3% dissimilarity. Chao, Shannon, Simpson and Phylogenetic diversity (Pd) were calculated to estimate the richness and diversity of the fungal community of each sample in two times (winter and summer) separately.

The fungal OTUs were transformed into text formatting, and the text was uploaded to the FUNGuild Taxonomic Function (http://www.stbates.org/guilds/app.php) for fungal functional prediction [25].

## 3. Result

### 3.1. Soil Physicochemical Properties

With the increased years of *C. chinensis* cultivation, the total content of nitrogen, phosphorus and organic matter significantly increased in both winter and summer seasons. Soil organic matter varied from 11.16 to 36.04 g kg^−1^ in the winter and from 8.35 to 33.90 g·kg^−1^ in summer. The soil of the five-year (Y5) fields had the highest content of organic matter in winter and summer (36.04, 33.90 g·kg^−1^ respectively), and its lowest in the one-year (Y1) fields (11.16, 8.35 g·kg^−1^). The organic matter contents were significantly lower in the continuous cropping fields than fallow fields (FF). The soil total content of nitrogen varied from 0.99 to 3.27 g·kg^−1^ in winter and from 1.02 to 3.13 g kg^−1^ in summer, among which the five-year (Y5) soil had the highest content in winter, and the FF had the highest content in summer (3.27, 3.13 g·kg^−1^), while the one-year (Y1) fields had the lowest content in both winter and summer. 

The total content of phosphorus was higher in five-year (Y5) continuously cropped fields in both winter and summer (4.18, 4.18 g kg^−1^ respectively), and the lowest content in fallow fields (FF) (1.17, 1.47 g·kg^−1^). Furthermore, the pH was slightly acidic, and varied from 4.49 to 5.84 in winter and from 5.59 to 6.48 in summer, being the highest value in one-year (Y1) and the lowest value in five-year (Y5) in two seasons (Table 1).

### 3.2. Biodiversity

#### 3.2.1. Alpha Diversity

Sequences were randomly selected from the modified sequences, and the number of sequences was plotted against the number of OTUs they represent to build the rarefaction curves (Figure S1). The curves trended to be flat as the number of sequences increased, and the upper limit of OTUs for one-year (Y1), three-year (Y3), five-year (Y5) and fallow fields (FF) were 318, 257, 250 and 174, respectively, in the winter season, and 357, 321, 307 and 246 in one-year (Y1), three-year (Y3), five-year (Y5) and fallow fields (FF) respectively, in the summer season. Thus, we concluded that our sequencing data were suitable for analysis, and that more sequencing data would not give many more OTUs. The upper limit of OTUs for both three-year (Y3) and five-year (Y5) was lower than for one-year (Y1) cropped fields, indicating that continuous cropping caused a decrease in the community diversity, and it was evident by after one year of constant cropping, which corresponded with the diversity indices. Rarefaction curves of OTUs were clustered at < 97% sequence identity for the four different *C. chinensis* rhizosphere soil from 1, 3 and 5 years continuous cropping of one sample to the control fallow field.

**Figure 1:**
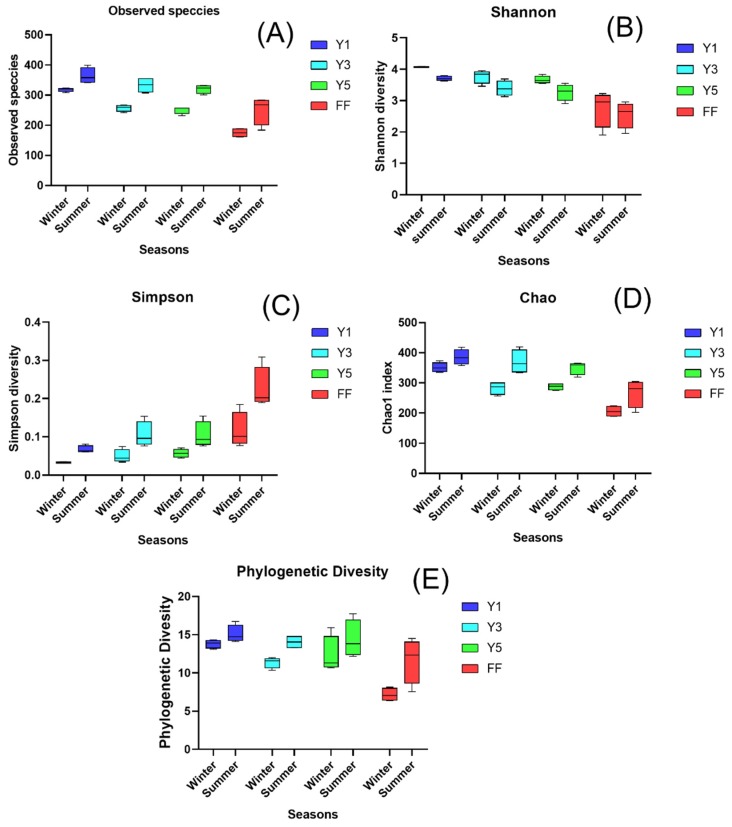
Alpha diversity indices of the fungal community in *C. chinensis* rhizosphere soils and fallow fields. Observed species richness (**A**), Shannon diversity index accounting for species abundance and evenness of distribution (**B**), Inverse Simpson diversity (**C**), Chao that estimates the actual species richness of sample (**D**), and the phylogenetic diversity (**E**) of FF, that is, the fallow field soil, where Y1 represents when one-year *C. chinensis* cultivated the soil, Y3 where three-year *C. chinensis* cultivated the soil, and Y5 indicates that five-year *C. chinensis* cultivated the soil. There were four independent replicates of each treatment.

Alpha diversity indices Chao, Shannon, Simpson, Observed OTUs and Phylogenetic diversity (Pd), were used to reveal the diversity and richness within samples. The alpha diversity except for the Simpson diversity indices was considerably higher in the one-year fields (Y1) as compared to the three-year (Y3) and five-year (Y5) fields in winter and summer. The alpha diversity indices showed higher in the winter seasons as compared with summer seasons. The diversity was lower in the fallow fields (FF) as compared to the cultivated fields (Y1, Y3, and Y5). Among the cultivated fields, the observed OTUs were significantly higher in the one-year (Y1) (Table S1 and Figure 1).

#### 3.2.2. Beta Diversity

**Figure 2:**
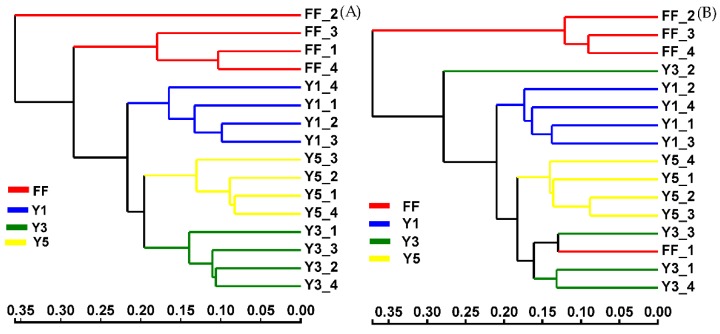
Bray–Curtis dissimilarity hierarchical cluster tree of soil fungal community; in winter (**A**) and summer (**B**); FF: fallow field soil; Y1: one-year *C. chinensis* cultivated the soil; Y3: three-year *C. chinensis* cultivated the soil; and Y5: five-year *C. chinensis* cultivated the soil.

Beta-diversity using the Bray–Curtis distance matrix revealed that the fungal communities in the soil samples collected from the same fields in the same year were more similar to each other than the corresponding soil samples of different years. For example, the fungal communities of the soil from the one-year cropping fields (Y1) were clustered differently than the three-year (Y3) and five-year (Y5) of cropping fields. However, the microbial communities of the three-year (Y3) and five-years (Y5) were more similar to each other than those microbial communities of the one-year (Y1) (Figure 2). 

**Figure 3:**
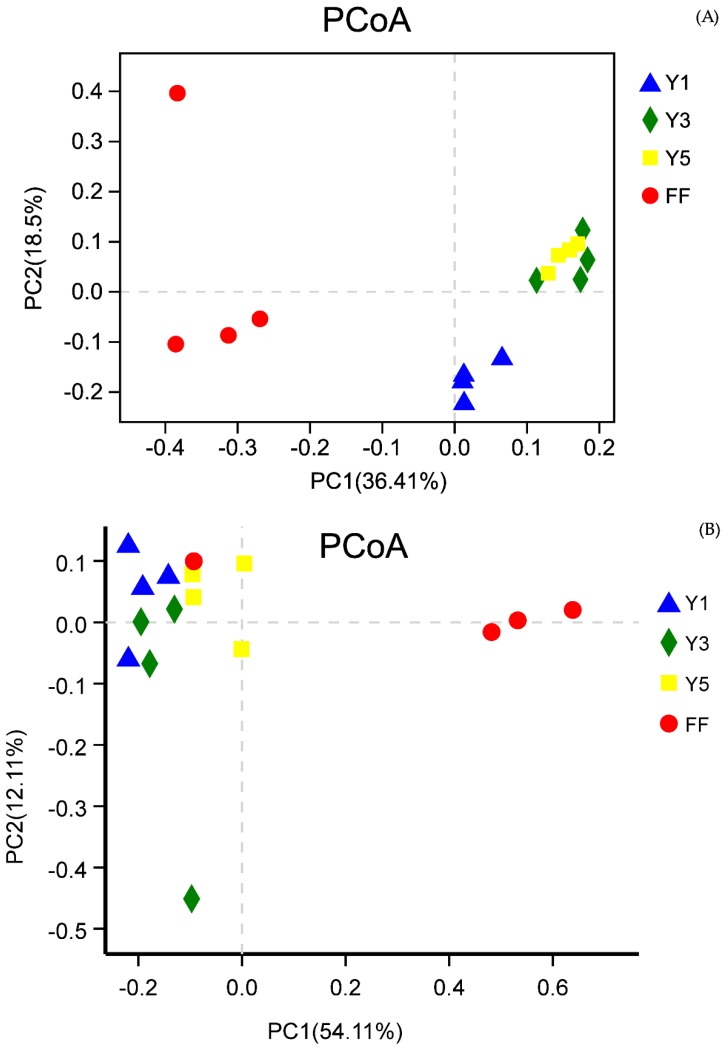
Principle coordinate analysis (PcoA); in winter (**A**) and summer (**B**); FF: fallow field soil; Y1: one-year *C. chinensis* cultivated the soil; Y3: three-year *C. chinensis* cultivated the soil; and Y5: five-year *C. chinensis* cultivated the soil.

Principle coordinate analysis (PCoA) based on UniFrac distances, i.e., unweighted and weighted, revealed that the rhizospheric fungal communities of the *C. chinensis* in the different years clustered separately in the summer season of the cropping years; however, the rhizospheric fungal communities of the three-year (Y3) and five-years (Y5) were grouped in the winter season. The result indicates that the fungal communities of the three-year (Y3) and five-years (Y5) are more similar to each other than the fungal communities of the one-year (Y1) are. When cultivated fields are compared with the fallow fields (FF), the fallow fields (FF) fungal communities clustered in different axis. The first two axes explain 36.41% and 18.5% of the total variation for the winter data and 54.11% and 12.11% for summer soil sample data. Additionally, in the winter, the fungal communities in the one-year (Y1) cultivated field were separated from the other two three-years (Y3) and five-years (Y5) cultivated soil samples by PC2, and the cultivated soil samples were separated by PC1 from the fallow field (FF). Furthermore, three-year (Y3) and five-year (Y5) field soil samples in winter had the most similar fungal community memberships (Figure 3). 

Venn diagrams can show the similarity and overlap of the species between different samples directly. There were 17, 3, 5 and 2 unique species which were present in the one-year (Y1), three-year (Y3), five-year (Y5) and fallow (FF) fields in winter, respectively, while 20, 7, 10 and 21 unique species were present in the summer sampling time, respectively. In winter, 100 (7.65%) common species were shared among the four groups of soil samples, which is the one-year (Y1) cultivated *C. chinensis* field had in summer the highest species number. Whereas, in summer, 123 (7.36%) species were shared among the samples. 

**Figure 4:**
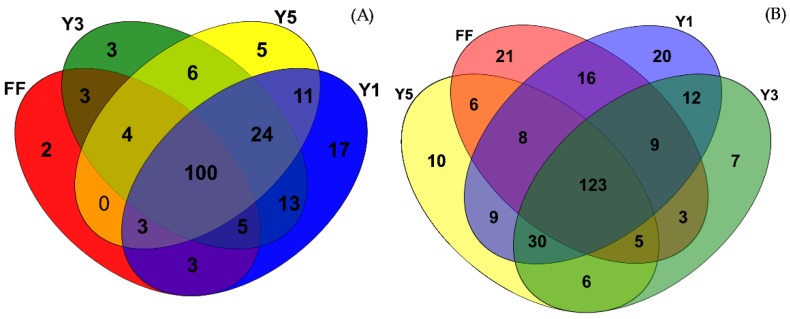
Venn diagrams showed shared and unique species of fungi in winter (**A**) and summer (**B**); FF: fallow field soil; Y1: one-year *C. chinensis* cultivated the soil; Y3: three-year *C. chinensis* cultivated the soil; Y5: five-year *C. chinensis* cultivated the soil.

A similar result in winter, the one-year (Y1) cultivated *C. chinensis* field had the highest species number. In winter and summer, the cultivated fields had a more significant amount of species compared with the fallow fields (Figure 4). These indicate that continuous cropping decreased the community diversity of fungi in rhizospheric soil.

### 3.3. Description of Fungal Communities

**Figure 5:**
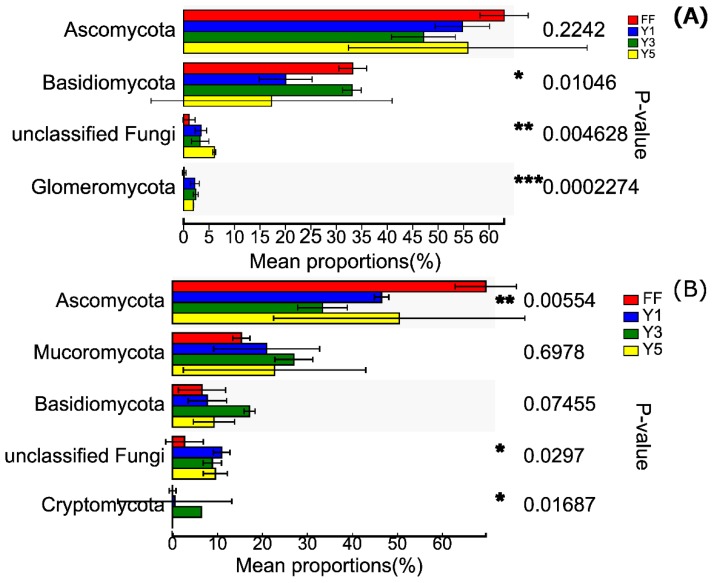
Multiple comparisons between the relative abundance of eight top fungal phyla: in winter (**A**) and summer (**B**); FF: fallow field soil; Y1: one-year *C. chinensis* cultivated the soil; Y3: three-year *C. chinensis* cultivated the soil; Y5: five-year *C. chinensis* cultivated the soil. * shows significant difference (p-value<0.05), ** shows significant difference (p-value<0.01), *** shows significant difference (p-value<0.001).

The OTUs present in these four samples and two seasons, including no-rank and unclassified, were divided into 36 phyla, 114 families and 131 genera in the winter season, and 46 phyla, 172 families and 209 genera in the summer season. Among the 36 phyla, the *Ascomycota* (55.20% of all sequence reads), *Basidiomycota* (25.94%), unclassified fungi phyla (3.46%) and *Glomeromycota* (1.68%) were the top four fungal phyla in winter. Whereas, among the 46 phyla, the *Ascomycota* (49.98% of all sequence reads), *Mucoromycota* (21.48%), *Basidiomycota* (10.16%) and unclassified fungi (7.99%) were the four most predominate fungal phyla in the summer season (Figure 5).

Multiple comparisons results showed that the mean proportions of *Ascomycota* were significantly higher in fallow fields (FF) (69.67%) in summer. The relative abundance of *Basidiomycota* was significantly higher in fallow fields (FF) (33.24%) in winter, but not significantly higher in three-year (Y3) cropping fields (17.14%) in summer. Compare to the fallow fields (FF), the mean proportion of *Glomeromycota* was markedly higher in one-year (Y1), followed by three-year (Y3) and five-year (Y5) cropping fields in winter. The relative abundance of *Mucoromycota* was considerably higher in the three-year (Y3) (26.98%) than the other fields in summer (Figure 5).

In the class taxonomic level, Sordariomycetes, Tremellomycetes, Lecanoromycetes and Agaricomycetes in winter, and Sordariomycetes, Mucoromycota, Leotiomycetes, Tremellomycetes and Agaricomycetes in summer were the most abundant fungal class (relative abundance > 2%) among the 20 most abundant fungal classes. The relative abundance of Sordariomycetes was significantly higher in five-year (Y5) cropping fields (35.94, 35.57%, respectively) in winter and summer (Table S2).

At the genus taxonomic level, *Boeremia, Mrakia, Hypocrea, Fusarium,* and *Pseudoplatyophyra* were identified in winter and *Chaetomium*, *Saitozyma, Heterocephalacria, Boeremia* and *Colpoda* were defined in summer. Among these genera, the relative abundance of *Fusarium* and *Hypocrea* were increased with the increase of cropping years from 0.46%, 1.10%, respectively, in one-year (Y1) to 1.60%, 3.67% in five-year (Y5) in winter, but in summer seasons the *Fusarium* and *Hypocrea* genera were not identified (Table S3).

### 3.4. Functional Prediction of Fungal Communities

**Figure 6:**
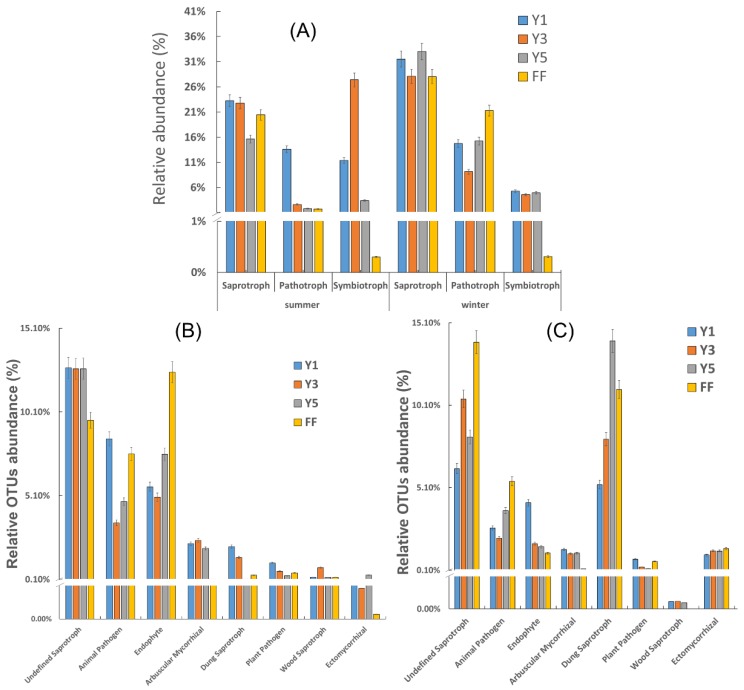
The relative abundance of three trophic modes in rhizosphere soil (**A**) relative abundance functional guild winter (**B**) and summer (**C**); FF: fallow field soil; Y1: one-year *C. chinensis* cultivated soil; Y3: three-year *C. chinensis* cultivated soil; Y5: five-year *C. chinensis* cultivated soil.

FUNGuild classified three main trophic modes (Pathotroph, Saprotroph and Symbiotroph), including eight functional guilds in our samples. The OTUs without any assigned functions dominated sequence richness (55.96%) in winter and (62.28%) in summer. In assigned OTUs with function, the saprotroph was the dominant trophic type in winter and summer, except in the three years (Y3) block in summer that was dominated by symbiotroph (27.41%). Besides, pathotroph was the dominant fungal trophic mode in one-year (Y1) (13.60%) in summer and fallow fields (FF) (21.27%) in winter. Moreover, the pathotroph trophic mode showed an increasing trend in winter and a decreasing trend in summer with the increasing number of cultivation years. Conversely, the relative OTUs’ abundance of symbiotroph was decreased from 10.62% in winter to 3.76% in summer. Symbiotroph becomes the maximum abundance group in Y3 (27.41%) in summer and one-year (Y1) (5.25%) in winter (Figure 6A).

In the functional guild, Undefined Saprotrophs, Animal Pathogen, Dung Saprotroph and Plant Pathogen were detected in higher abundances in one-year (Y1) (12.73%, 8.48%, 2.06% and 1.09%, respectively), in winter. However, in summer, Undefined Saprotroph, Animal Pathogen and Ectomycorrhizal had higher Relative OTUs abundances in FF 32.93%, 5.49% and 1.41%, respectively (Figure 6B,C).

### 3.5. Effect of Environmental Factors on Fungal Communities

**Figure 7:**
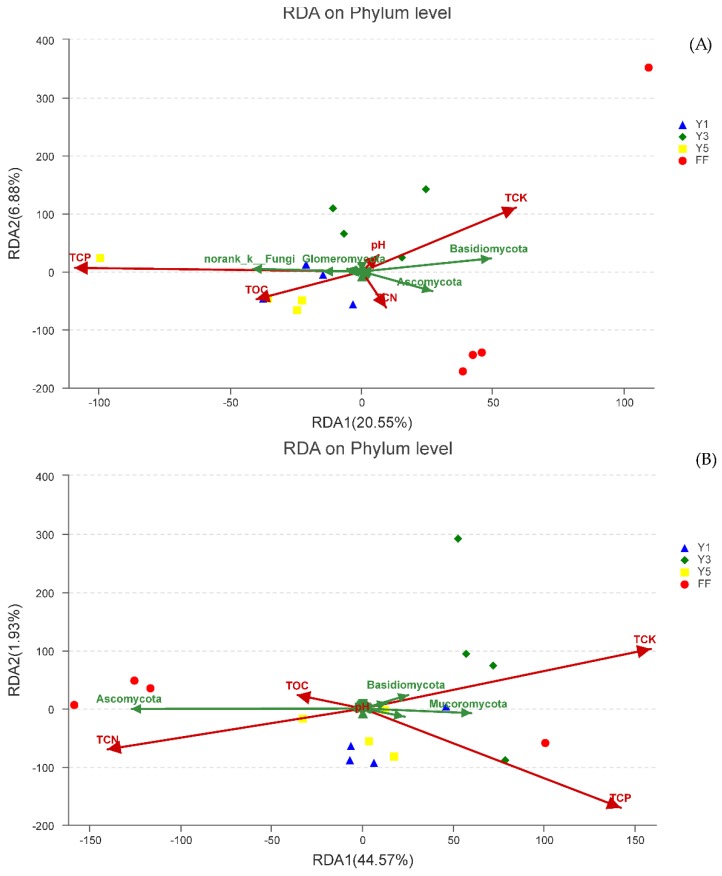
The length and angle of lines represent redundancy analysis (RDA) of the abundant fungal phyla and environmental variables in winter (**A**) and summer (**B**); Correlations between RDA axes and the environmental variables. Correlations between RDA axes and the rhizosphere fungal phyla are represented by words (i.e., the fungal phylum names). FF: fallow field soil; Y1: one-year *C. chinensis* cultivated soil; Y3: three-year *C. chinensis* cultivated soil; Y5: five-year *C. chinensis* cultivated soil; TCN: total content of Nitrogen; TCP: total content of phosphorus; TCK: total content of potassium; TOC: total organic carbon and pH.

Redundancy analysis (RDA) of the dominant phyla data and soil physiochemical properties revealed variations in fungal community structure in the soil samples. The first two RDA components (RDA1 and RDA2) explain 20.55% and 6.88% of the total variance, respectively, in winter, and 44.57% and 1.93% (RDA1 and RDA2), in summer. No significant trends were observed between the soil environmental factors and the abundances of the top 30 most common OTUs across the 12 soil samples from four different fields. However, if we examine the phylum-level relative abundances, the strong influence of soil environmental variables on overall fungal community composition is evident (Figure 7). To investigate the significances of the effects of soil environmental factors on bacterial community composition, we calculated the r^2^ and *p*-values. Among these soil environmental factors, the total content of phosphorus (TCP) showed both the highest r^2^ value (r^2^ = 0.4375, *p*-values = 0.011) in winter, indicating that TCP had the highest effect on fungal community composition.

**Figure 8:**
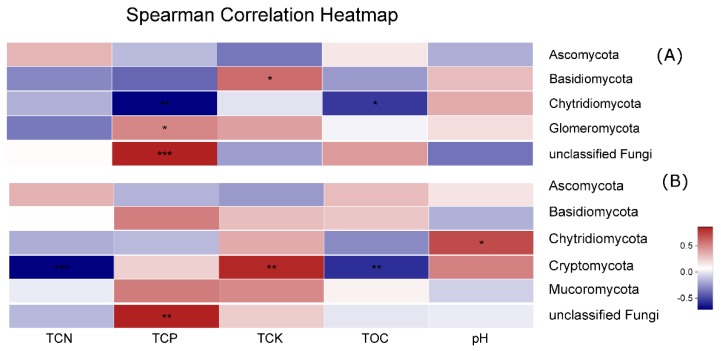
The heatmap of the correlation between fungal phyla and physicochemical characteristics of rhizospheric soil (**A**) in winter and (**B**) in summer. This heatmap was created according to the result of Spearman’s correlation analysis. Positive relationships are represented in red, while negative relationships are represented in blue. The significant correlations are presented as asterisks (*, *p* < 0.05; **, *p* < 0.01; ***, *p* < 0.001).

Moreover, Spearman’s correlation coefficient was used to evaluate the relationship between soil physicochemical properties and fungal phyla abundance (Figure 8). The *Basidiomycota* abundant showed a positive correlation (r = 0.594) with TCK in winter, *Glomeromycota* was positively correlated (r = 0.5) with TCP in winter, *Chytridiomycota* showed a negative relationship with TCP and TOC (r = 0.717, r = 0.545) respectively, in winter. *Cryptomycota* phyla relative abundances were negatively correlated with TCN and TOC and positively correlated with TCK in summer.

## 4. Discussion 

Continuous cropping obstacles are caused by complex factors within the soil–crop–microbial system, and these need to be resolved through a combination of multiple methods. There are many factors related to continuous cropping obstacles, such as soil enzyme activity, soil physical and chemical properties and root exudates [26]. Numerous studies have reported that continuous cropping led to the accumulation of organic acids and phenolic acids secreted by roots, promoting the growth of pathogenic microorganisms and further affecting the structure of the rhizospheric soil microbial community [6]. About 70% of medicinal plant species having tuber roots with different degrees are affected by continuous cropping obstacles such as *Rehmannia glutinosa*, *Pseudostellaria heterophylla,* and *Panax notoginseng*, etc. [18,27]. Our results showed that the continuous cropping of *C. chinensis* and seasons significantly affected soil properties (Table 1). In this study, the total content of nitrogen, phosphorus and organic matter all showed an increasing trend with the increase of cultivation years in winter and summer, which may result from the misuse of inorganic fertilizers [28,29]. Moreover, a similar result was also reported by Tan et al. [30] in continuous *P. notoginseng* cropping, which could also be attributed to a long-term oversupply of chemical fertilizer [31]. Lower pH was observed in the continuous cropping of *C. chinensis* fields, and possible reasons may be the long-term application of nitrogen fertilizer and the aggregation of allelochemicals [32], leading to soil acidification in *C. chinensis* fields [28,33]. Another possible reason for acidification in *C. chinensis* continuous cropping systems may be the different bioactive catechins produced by roots, leaves and other residues, that are used in the fields.

Microbial community richness and diversity play a critical role in soil quality, function and productivity [2]. In this study, we found that the continuous cropping of *C. chinensis* resulted in a considerable change in soil fungal community diversity and richness. The observed species richness, Shannon diversity index, Chao1 and phylogenetic diversity indices showed a decreasing trend with the increase in the number of crop years in winter and summer, and were significantly higher than results from FF (Figure 1). Similarly, Song et al. [2] have found that the number of OTUs and the Shannon diversity index declined consistently with the increase in the number of continuous cropping years of *C. chinensis*. Similar results have also been observed in Notoginseng [34], tea [33], coffee [35], soybean [36], etc. The decreases in fungal diversity and richness have been recognized as an essential threat to the ecosystem, resulting in loss of soil function [37,38], which may arise in reduced *C. chinensis* production under continuous cropping practices.

The result of Hierarchical clustering analysis based on the Bray–Curtis distance and UniFrac-weighted principal coordinate analysis (PCoA) in this study, demonstrated that the continuous cropping of *C. chinensis* had strong effects upon the soil fungal community structure and showed apparent variations in the fungal community (Figure 2 and Figure 3). This result was consistent with the outcome of Xiong et al. [28], that the continuous cultivation of vanilla had a significant influence on alterations in the fungal community structure. This phenomenon has also been observed for many other perennials and annual crops such as soybean [36,39], *Panax notoginseng* [30], potato [40], coffee [35], peanut [14] and tea [35] continuous cropping systems. Thus, we can hypothesize that soil microbial communities could be affected by continuous cropping. 

In the agro-ecosystem, the soil microbial community as a soil quality indicator plays an essential role in nutrient cycling and organic matter dynamics. Amendments in the composition of soil microbial communities or microbial biomass resulted in changes in soil quality [30,41]. In the present study, we found that *Ascomycota* (according to 55.20% and 49.98% RA, respectively) was the most abundant fungal phylum in the samples in winter and summer (Figure 5). Moreover, the relative abundance of *Ascomycota* was increased in five-year (Y5) continuous cropping compared with one-year (Y1), but compared with fallow fields (FF), were decreased. *Basidiomycota* (according to 25.94% RA) was the second most abundant fungal phylum in winter, and showed a decreasing trend in five-year (Y5) cropping fields compared with one-year (Y1) and fallow fields (FF). *Mucoromycota* (according to 21.48% RA) was a dominant phylum, and showed an increasing trend in five-year (Y5) continuous cropping compared with one-year (Y1) and fallow fields (FF) in summer. These findings generally agree with previous studies reporting that Ascomycota and Basidiomycota were the top two most abundant phyla in continuous cropping [14]. Furthermore, similar results have also been found in forest soils and other soil types in nature [42,43]. *Sordariomycetes* (according to 22.68% RA), *Tremellomycetes* (according to 17.32% RA) in winter and *Sordariomycetes* (according to 24.20% RA), *Mucoromycota* (according to 19.71% RA) in summer were also dominant fungal classes. At the genus taxonomic level, the relative abundance of *Fusarium*, *Hypocrea* were increased with the increase of cropping years from 0.46%, 1.10%, respectively, in one-year (Y1) to 1.60%, 3.67% in five-year (Y5) in winter. Similarly, Song et al. [44] observed that the continuous cropping of *C. chinensis* resulted in an increased relative abundance of *Fusarium* and *Hypocrea.*

FUNGuild is a useful database for the function of members within the fungal community, and could provide information on the functional roles of fungi from the way of trophic guilds rather than resolving the purpose from individual taxa [45,46]. The undefined saprotroph dominated the functional guilds in winter and summer (Figure 6), which could play central roles in organic decomposition [25]. Furthermore, the result was consistent with Wei et al. [47] animal pathogen and plant-pathogen are the significant functional guilds, and are mostly intracellular and parasitize plants more widely than animals [48]. However, in the present study, the relative abundance of the plant pathogens showed significantly lower proportions than that of animal pathogens in winter and summer (Figure 6). Animal pathogens showed a lower percentage in five-year (Y5) continuous cropping compared with one-year (Y1) in winter, but a higher proportion in five-year (Y5) compared with one-year (Y1) in summer. On the other hand, the relative proportion of plant pathogen showed a lower percentage of five-year (Y5) compared with one-year (Y1) in winter and summer. This study demonstrated that the similar function of these OTUs had different distributions, suggesting the essential roles of rare fungal sub-community in standard features.

Soil physicochemical properties have an essential role in structuring microbial communities [49]. According to some reported studies, Soil properties could be a significant factor in controlling microbial community structure [43,50,51]. RDA analyzed the impacts of the total content of nitrogen, phosphorus and potassium, soil pH and organic matter content on the fungal community. This result was in agreement with some previous studies in which the rhizospheric soil fungal community composition and structure were significantly altered based on the number of continuous cropping years [2,30,36].

Determining the correlation between the diversity of the soil fungal community and the soil environmental factors could provide a better understanding mechanisms of continuous cropping obstacles [28]. In this study, we found that the *Basidiomycota* abundance showed a positive correlation with TCK in winter, *Glomeromycota* was positively correlated with TCP in winter, and that *Chytridiomycota* showed a negative relationship with TCP and TOC in winter. The relative abundance of the *Cryptomycota* phylum was negatively correlated with TCN and TOC, and positively correlated with TCK in summer (Figure 8). Changes in fungal community structure in response to the continuous cropping of *C. chinensis* could not be only referred to deviations in soil chemical properties, but may also be under long-term influences of *C. chinensis* plant root exudates or residues [52], which could not be determined in the range of this study.

## 5. Conclusions

Our results show that the continuous cropping of *C. chinensis* led to significant ascends in the total content of nitrogen, phosphorus and organic matter, and declines in pH and fungal community richness and diversity. Furthermore, in this study, we found that cropping years had a significant influence on soil fungal community composition and structure. In addition, considerable variation was found among the fungal trophic guilds group in soil samples, which were affected by continuous cropping years. Thus, these results provide a platform for developing sustainable agricultural research to enhance microbial activity and boost *C. chinensis* production in continuous cropping soils, which is vital for *C. chinensis* production in China. 

## Figures and Tables

**Table 1:** Physiochemical characteristics of rhizospheric soil in winter and summer.

Seasons	Samples	TCN (g/kg)	TCP (g/kg)	TCK (g/kg)	TOM (g/kg)	pH
Winter	FF	2.66 ± 0.33ab	1.17 ± 0.04d	21.95 ± 0.58c	21.70 ± 0.64b	4.88 ± 0.08b
	Y1	0.99 ± 0.16c	1.66 ± 0.03c	27.88 ± 1.13b	11.16 ± 1.45c	5.84 ± 0.33a
	Y3	2.09 ± 0.26b	2.82 ± 0.44b	30.17 ± 1.03a	21.62 ± 1.57b	5.11 ± 0.32b
	Y5	3.27 ± 0.68a	4.18 ± 0.23a	9.27 ± 0.22d	36.04 ± 3.33a	4.49 ± 0.01c
Summer	FF	3.13 ± 0.91a	1.47 ± 0.35c	21.86 ± 2.10b	21.44 ± 1.23b	6.20 ± 0.21b
	Y1	1.02 ± 0.28b	1.97 ± 0.27bc	31.21 ± 2.97a	8.35 ± 1.12c	6.48 ± 0.25a
	Y3	1.52 ± 0.78b	2.35 ± 0.71b	31.86 ± 1.71a	19.23 ± 0.68b	6.09 ± 0.13b
	Y5	2.68 ± 0.48a	4.18 ± 0.38a	9.30 ± 1.17c	33.90 ± 4.49a	5.59 ± 0.10c

TCN: total content of Nitrogen; TCP: total content of phosphorus; TCK: total content of potassium; TOM: total organic matter and pH. FF: fallow field soil; Y1: one-year *C. chinensis* cultivated the soil; Y3: three-year *C. chinensis* cultivated the soil; Y5: five-year *C. chinensis* cultivated the soil. Values are means ± standard deviation (n = 4). a,b,c,d; means followed by the same letter for a given factor are not significantly different (*p* < 0.05; Tukey’s HSD test).

## Supplementary Materials

The following are available online at https://www.mdpi.com/2223-7747/9/2/244/s1, Figure S1: Rarefaction curves of soil fungal communities based on observed at 97% sequence similarity level in four times soil samples from *C. chinensis* fields. The X-axis represents the number of randomly selected sequences; the Y-axis represents the number of species. FF, Y1, Y3 and Y5 represent the non-cultivated field, one-year cultivated field, three-years cultivated field, and five-years cultivated field, respectively. A. winter B. summer. Table S1: Fungal Alpha diversity. TCN: total content of Nitrogen, TCP: total content of phosphorus, TCK: total content of potassium, TOC: total organic carbon and pH. FF: fallow field soil, Y1: one-year *C. chinensis* cultivated soil, Y3: three-year *C. chinensis* cultivated soil, Y5: five-year *C. chinensis* cultivated soil. Values are means ± standard deviation (n = 4). Means followed by the same letter for a given factor are not significantly different (*p* < 0.05; Tukey’s HSD test). Table S2: The relative abundance (<0.01%) of fungal groups of each sample at the class level. Table S3: The relative abundance (%) of fungal groups of each sample at the genus level in winter and summer season.

## Abbreviations

NGS	Next-Generation Sequencing
RDA	Redundancy Analysis
PcoA	Principle Coordinate Analysis
OTUs	Operational Taxonomic Units
